# Intra-fraction displacement of the prostate bed during post-prostatectomy radiotherapy

**DOI:** 10.1186/s13014-020-01743-9

**Published:** 2021-01-22

**Authors:** Linda J. Bell, Thomas Eade, George Hruby, Regina Bromley, Andrew Kneebone

**Affiliations:** 1 Northern Sydney Cancer Centre, Radiation Oncology Department, Royal North Shore Hospital, St Leonards, NSW 2065 Australia; 2 Northern Clinical School, University of Sydney, St Leonards, NSW 2065 Australia

**Keywords:** Intra-fraction motion, Prostate bed, Post-prostatectomy, Radiotherapy, IGRT

## Abstract

**Background:**

To measure intra-fraction displacement (IFD) in post-prostatectomy patients treated with anisotropic margins and daily soft tissue matching.

**Methods:**

Pre-treatment cone beam computed tomography (CBCT) scans were acquired daily and post-treatment CBCTs for the first week then weekly on 46 patients. The displacement between the scans was calculated retrospectively to measure IFD of the prostate bed (PB). The marginal miss (MM) rate, and the effect of time between imaging was assessed.

**Results:**

A total of 392 post-treatment CBCT’s were reviewed from 46 patients. The absolute mean (95% CI) IFD was 1.5 mm (1.3–1.7 mm) in the AP direction, 1.0 mm (0.9–1.2 mm) SI, 0.8 mm (0.7–0.9 mm) LR, and 2.4 mm (2.2–2.5 mm) 3D displacement. IFD ≥  ± 3 mm and ≥  ± 5 mm was 24.7% and 5.4% respectively. MM of the PB was detected in 33 of 392 post-treatment CBCT (8.4%) and lymph nodes in 6 of 211 post-treatment CBCT images (2.8%). Causes of MM due to IFD included changes in the bladder (87.9%), rectum (66.7%) and buttock muscles (6%). A time ≥ 9 min between the pre and post-treatment CBCT demonstrated that movement ≥ 3 mm and 5 mm increased from 19.2 to 40.5% and 5 to 8.1% respectively.

**Conclusions:**

IFD during PB irradiation was typically small, but was a major contributor to an 8.4% MM rate when using daily soft tissue match and tight anisotropic margins.

## Background

Radical prostatectomy is the most common treatment for localised prostate cancer [1]. Approximately one third of these patients will have a rising PSA post-surgery and should be considered for radiotherapy to the prostate bed [2].

Previously published work has quantified inter-fraction motion in post-prostatectomy patients [3], with inter-fraction motion found to be the largest in the upper portion of the prostate bed. To manage this daily setup error a new image guided radiotherapy (IGRT) technique, aligning to soft tissue daily using cone beam computed tomography (CBCT), and anisotropic planning target volume (PTV) expansion was developed [4].

However, these studies did not incorporate real-time matching nor did they assess intra-fraction displacement (IFD). The aims of this prospective study were to:

(i) Quantify the size, direction and frequency of IFD,
(ii) Measure the frequency of marginal misses as per the post-treatment CBCT,
(iii) Assess the causes of marginal miss observed in the post-treatment CBCT, and
(iv) Assess impact of prolonged treatment time on IFD in post-prostatectomy patients.

## Methods

Following local ethics review board approval we reviewed CBCT images taken before and after post-prostatectomy treatment in 46 patients.

All patients included in this study were treated with an anisotropic PTV expansion, including 5 mm in all directions in the lower prostate bed and 5 mm in all directions except anteriorly and posteriorly in the upper prostate bed, where a 10 mm expansion was used. All patients were matched daily to soft tissue using CBCT. Thirty-five patients were treated with 68 Gy in 34 fractions and 11 received 64 Gy in 32 fractions. Thirty patients (65.2%) had surgical clips in-situ which assisted daily IGRT. Prophylactic lymph node volumes were treated in 25 of the 46 patients. Forty-three patients were treated using volumetric modulated arc therapy (VMAT) with either 2 or 3 arcs and 3 were treated with 7 to 11 field intensity modulated radiotherapy (IMRT).

### Evaluation of the amount of IFD

The pre-treatment CBCT were matched with a daily soft tissue protocol as previous described [5]. As per department protocol, at completion of treatment on fractions 1–5 and then weekly, a post-treatment CBCT was acquired. The CBCT was reviewed offline as per the soft tissue matching protocol using Offline Review (Varian Medical Systems, Palo Alto, CA, USA).

Following completion of the treatment course, the matches completed online on all of the pre and post-treatment images taken during the first five fractions and one each subsequent week, were reviewed by a single observer using the Offline Review software. If setup error caused a marginal miss on the pre-treatment CBCT, the images from that fraction were removed from the IFD calculations. IFD was calculated as the difference between the pre and post-treatment match. The IFD in the anterior–posterior (AP), superior–inferior (SI), and left–right (LR) directions was calculated. A 3-dimensional displacement value was also calculated using the root sum of squares method

$${\text{3D Displacement}} = \sqrt {x^{2} + y^{2} + z^{2} }$$

where *x* = anterior–posterior IFD, *y* = superior–inferior IFD, z = Left–right IFD.

### Marginal miss detected on post-treatment imaging

**Fig. 1:**
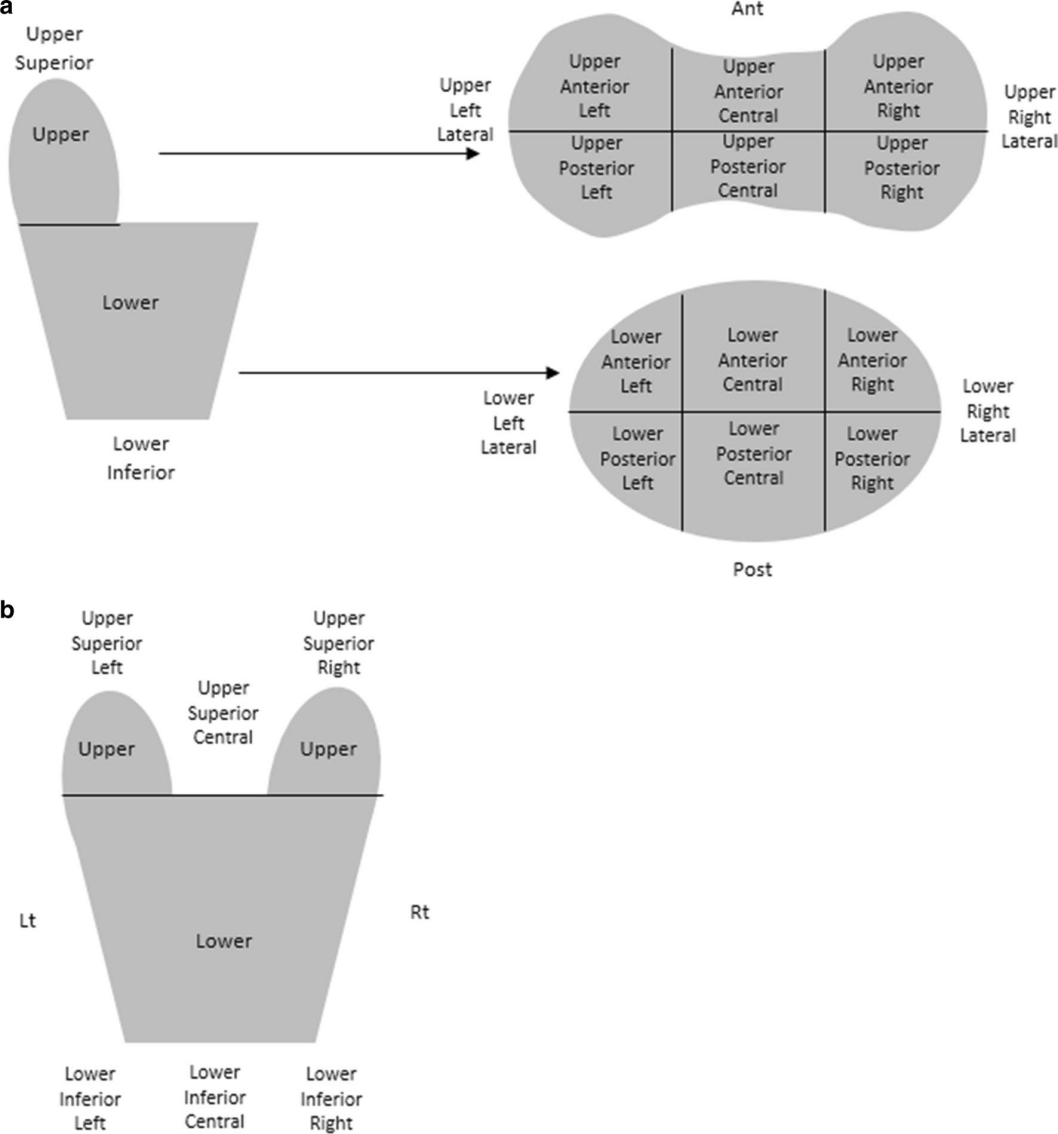
Schematic diagram of the division of the prostate bed. Diagram of the location descriptions of the regions of the **a** prostate bed and **b** the area adjacent to the superior and inferior aspects of the prostate bed

Marginal miss was defined as any soft tissue and/or surgical clips contoured initially within the clinical target volume (CTV) on the planning CT scan being located outside the PTV on the post-treatment CBCT. If a marginal miss was detected during the retrospective review, the region of the treatment area at risk was documented and the amount of tissue not covered was measured. The naming of the regions of the prostate bed are displayed in Fig. 1. The amount of tissue missed was measured as the largest amount of tissue outside the PTV which was contoured in the CTV measured by the measuring tool in Offline Review.

### Causes of marginal miss on post-treatment imaging

Each CBCT image with a marginal miss was assessed to determine its cause. This was achieved by a review of differences between the pre and post-treatment image. The differences were categorised to determine which issue provided the largest potential for causing marginal miss. The categories included rectal gas moved, rectal filling moved, rectum larger than at simulation, bladder filled, bladder larger than at simulation, bladder smaller than at simulation, buttocks cheeks tensed, and buttocks cheeks relaxed.

### Time between pre and post-treatment imaging effect on IFD

Using Offline Review the amount of time between the pre and post-treatment CBCT images was measured and compared with the 3D IFD and marginal miss recorded for the first 46 patients to determine how treatment time impacts on motion. The images were grouped into 2 cohorts which were determined by calculating the mean time between the pre and post-treatment CBCT image acquisition then rounding up to a full number which would be easy to remember if these values were used in a clinical setting. The number of images in each of these cohorts that demonstrated a 3D displacement ≥ 3 mm, ≥ 5 mm, or a prostate bed marginal miss were calculated to assess predictability and correlation was calculated.

Microsoft Excel (Microsoft Corporation, Redmond, WA, USA) and GraphPad Prism (GraphPad Software, La Jolla, CA, USA) were used to conduct statistical analyses on the collected data.

## Results

A total of 46 patients and 408 post-treatment CBCT images were reviewed which included 221 images with lymph node volumes. Pre-treatment misalignment was detected on 16 pre-treatment images and the associated post-treatment images were removed from all analysis, leaving 392 post-treatment CBCT displaying the prostate bed and 211 with lymph node volumes. The mean (95% CI) of the bladder and rectum volume at simulation was 295 cc (253.8 cc to 336.2 cc) and 68.85 cc (62.81 cc to 74.88 cc) respectively for the 46 patients.

### Evaluation of the amount of IFD

**Fig. 2:**
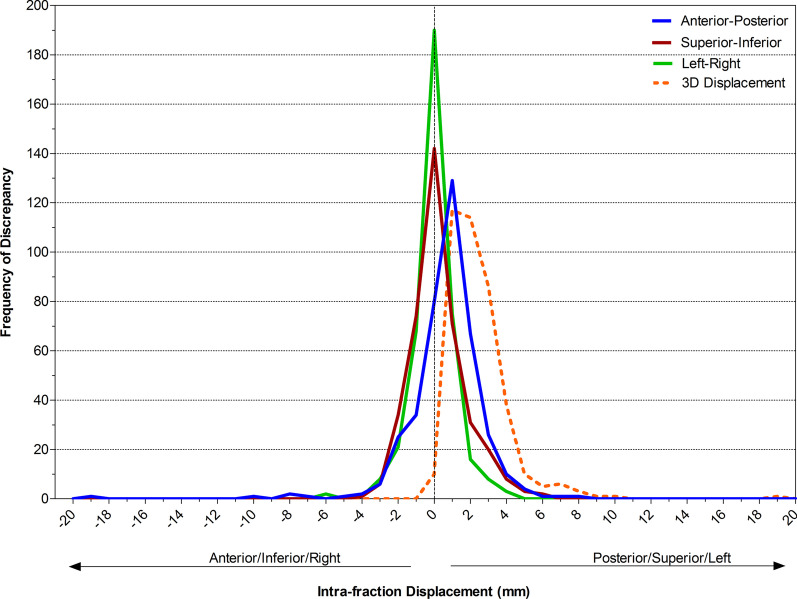
Frequency and magnitude of intra-fraction displacement. The intra-fraction displacement frequency and magnitude is displayed for the AP (blue), SI (red), LR (green), and 3D displacement (orange) directions

The frequency and magnitude of IFD detected on post-treatment imaging is display in Fig. 2 in the AP, SI, LR, and 3D displacement directions. The motion varied in size and direction. The largest motion detected was 19.1 mm in the anterior direction.

IFD was most common in the posterior direction. The median (range) of IFD was 1.0 mm (0.1–19.1 mm) in the Anterior direction, 1.2 mm (0.1–8.4 mm) Posterior, 1.3 mm (0.1–5.6 mm) Superior, 1.0 mm (0.1–3.6 mm) Inferior, 0.7 mm (0.1–3.7 mm) Left, 0.9 mm (0.1–6.5 mm) Right, and 3D displacement 2.1 mm (0–19.3 mm).

The absolute mean (95% CI) IFD for the 46 patients was 1.5 mm (1.3–1.7 mm) in the AP direction, 1.0 mm (0.9–1.2 mm) SI, 0.8 mm (0.7–0.9 mm) LR, and 2.4 mm (2.2–2.5 mm) 3D displacement.

IFD ≥  ± 3 mm and ≥  ± 5 mm tolerances was also calculated (Table 1). IFD was ≥  ± 3 mm and ≥  ± 5 mm in either the AP, SI or LR direction in 20.9% and 3.6% of fraction respectively and 3D displacement of ≥  ± 3 mm and ≥  ± 5 mm was detected in 24.7% and 5.4% of fractions respectively. The largest percentage of fractions with IFD ≥  ± 3 mm and ≥  ± 5 mm occurred in the AP (9.9% and 2.0%) direction followed by the SI (6.4% and 1.0%) and LR (4.6% and 0.5%) directions.

**Table 1:** Infra-fraction displacement ≥ 3 mm and 5 mm

Direction of Displacement	Number of fractions (percentage of all fractions) with intra-fraction displacement ≥ 3 mm	Number of fractions (percentage of all fractions) with intra-fraction displacement ≥ 5 mm
Any Direction (AP, SI, LR)	82 (20.9%)	14 (3.6%)
Anterior—posterior	39 (9.9%)	8 (2.0%)
Superior—inferior	25 (6.4%)	4 (1.0%)
Left—right	18 (4.6%)	2 (0.5%)
3D displacement	97 (24.7%)	21 (5.4%)

Comparison of the number and percentage of all fractions where intra-fraction displacement was ≥ 3 mm or 5 mm tolerance in each direction and 3D displacement

*AP* anterior–posterior, *SI* superior–inferior, *LR* left–right, *3D* 3 dimensional, *mm* millimetre

### Marginal miss detected on post-treatment imaging

**Fig. 3:**
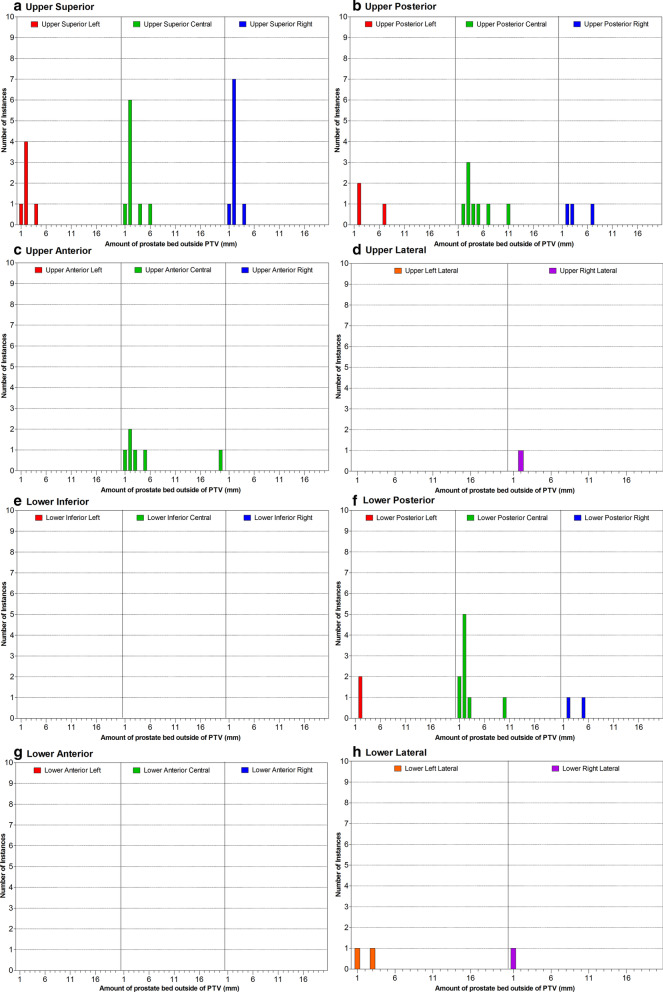
Location and amount of prostate bed marginal miss detected on the post-treatment images. The location and number of marginal misses of the prostate bed along with the amount of prostate bed not covered by the PTV is displayed. Each measure is displayed in the **a** upper superior, **b** upper posterior, **c** upper anterior, **d** upper lateral, **e** lower inferior, **f** lower posterior, **g** lower anterior, and **h** lower lateral location in the prostate bed

Marginal miss of the prostate bed was detected in 33 of 392 post- treatment CBCT (8.4%) and of the lymph node volume in 6 of 211 post-treatment CBCT image (2.8%). Figure 3 displays the location of prostate bed marginal misses detected on the post-treatment CBCT. The majority of misses occurred in the upper central portion of the prostate bed. The majority of the prostate bed marginal misses were small with 77% being ≤ 3 mm in size. Only 11% of the marginal misses measured > 5 mm.

### Frequency of marginal miss

Twenty-one of the 46 patients (45.7%) completed treatment without a marginal miss being detected. Twenty-five patients (54.3% of all patients) had at least one marginal miss detected during their treatment course. Sixteen of these patients only recorded one marginal miss, six patients recording two, two patients with three, and one patient with four marginal misses.

Marginal misses were categorised into prostate bed, lymph node, and both prostate bed and lymph node marginal misses. All lymph node marginal misses or combined prostate bed and lymph node marginal misses were detected in the first 5 fractions. Prostate bed marginal misses were seen throughout the treatment course with 19 occurring in the first 5 fraction and 13 from fraction 6 onwards. Of the 21 patients who had a marginal miss detected in the first 5 fractions, eight patients had another marginal miss detected during treatment, however 13 patients had no further marginal misses. Four patients had no marginal misses detected in the first 5 fraction but then had a marginal miss in at least 1 of the remaining fractions. This indicates that the marginal miss rate detected in the first five fractions is not a good predictor of the overall marginal miss rates seen during the treatment course.

### Causes of marginal miss on post-treatment imaging

**Fig. 4:**
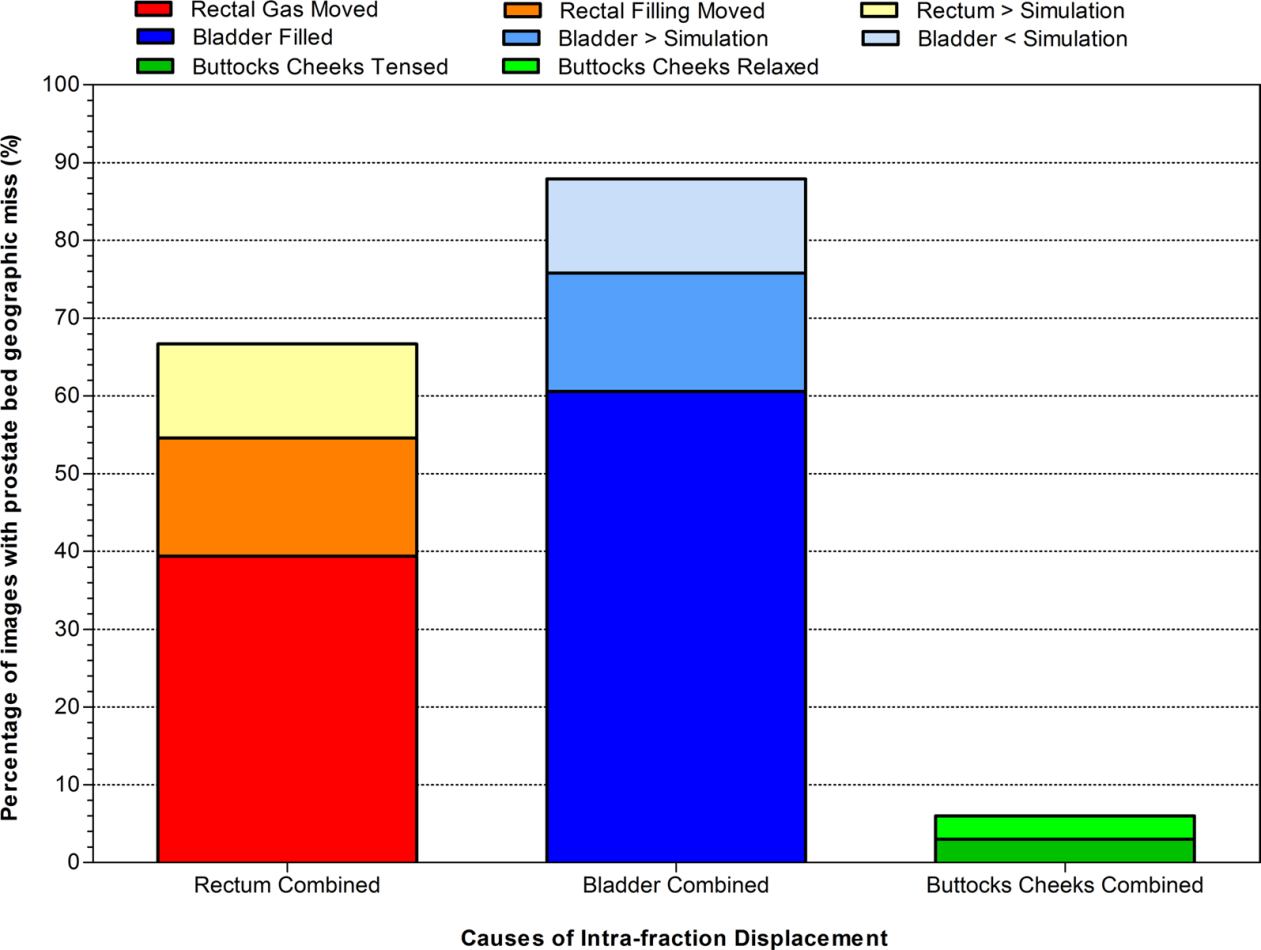
Causes of marginal miss detected on post-treatment images. The causes of marginal miss are displayed as a percentage of all post-treatment images that displayed a marginal miss of the prostate bed

The change between the pre and post-treatment CBCT were analysed for the fractions where prostate bed marginal miss was detected (Fig. 4). Multiple contributing factors were present in some images. Bladder (87.9% of images with marginal miss), rectum (66.7%), and buttocks cheek (6%) changes were identified as being the principle causes of IFD. Bladder filling during treatment occurred in 60.6% of the fractions and rectal gas moving in 39.4% of fractions where marginal miss was detected in the prostate bed.

### Time between pre and post-treatment imaging effect on IFD

**Fig. 5:**
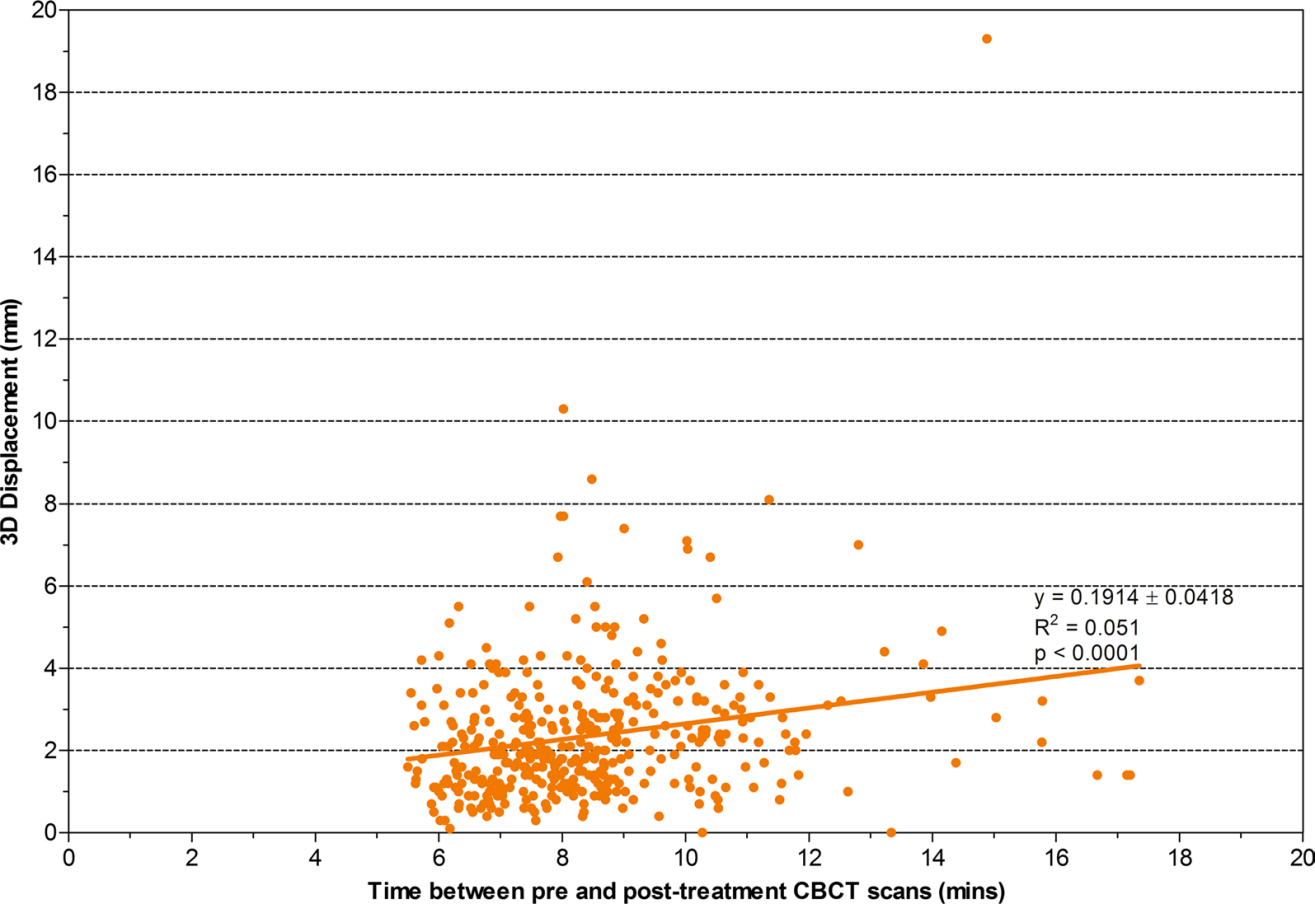
Correlation between time between pre and post-treatment imaging and IFD. The correlation between the time between the pre and post-treatment CBCT images is correlated against the IFD detected

Time between pre and post-treatment imaging ranged between 5.5 and 17.4 min with a mean of 8.5 min. Figure 5 illustrates the correlation between treatment time and 3D displacement. A significant positive correlation (*p* < 0.001) between pre and post-treatment imaging and 3D displacement was found. Time between pre and post-treatment imaging displayed an increase in movement ≥ 3 mm from 19.2% of images to 40.5% with times ≥ 9 min (Table 2). A time ≥ 9 min also resulted in an 11.7% marginal miss rate of the prostate bed. The variation in treatment time appeared a random event with treatments ≥ 9 min occurring in 85% of patients at some stage during their treatment course. The most common reason for prolonged treatment time was due to difficulty in patient set up or change in volume of rectum/bladder on CBCT compared to simulation though this has not been quantified. Two patients had a time of ≥ 9 min for all fractions with post-treatment imaging which was due to complex target volumes and treatment imaging techniques however these patients did not contribute high levels of motion to the findings with a mean 3D displacement of 2.4 mm and 1.9 mm respectively.

**Table 2:** Time between pre and post-treatment imaging effect on IFD

	Measurements	3D displacement ≥ 3 mm	3D displacement ≥ 5 mm	Prostate bed MM
Time between pre and post-treatment CBCT	≥ 9 min (n = 111)	45 (40.5%)	9 (8.1%)	13 (11.7%)
	< 9 min (n = 281)	54 (19.2%)	14 (5.0%)	20 (7.1%)

Impact of time on 3D intra-fraction displacement and marginal miss for the first 46 patients

*3D* 3 dimensional, *mm* millimetre, *MM* marginal miss, *n* number of patients, *CBCT* cone beam computed tomography, *min* minutes

## Discussion

With daily soft tissue matching and tight anisotropic margins we had a marginal miss rate of 8.4% for post prostatectomy radiotherapy in a real-time setting. Although total IFD was generally small (mean 1.5 mm), 24.7% had IFD > 3 mm and this rate of IFD significantly rises from 19.2 to 40.5% when treatment time is prolonged more than 9 min.

Comparing the results from the current study to previous studies is difficult due to the different equipment, methods and end points used to measure intra-fraction motion (Additional file 1). Two other studies used CBCT to measure intra-fraction motion with variations in the timing of acquisition [6, 7]. Our study demonstrated a larger overall amount of IFD of 2.4 mm compared with 0.4 mm [6], and a similar mean motion [7]. A number of groups used real-time monitoring to measure intra-fraction motion of the prostate bed either by introducing electromagnetic transponders (Calypso, Varian Medical Systems, Palo Alto, CA, USA) [8–10] or transperineal ultrasound (Clarity, Elekta, Stockholm, Sweden) (Additional file 1) [11, 12]. Most studies completed with real-time monitoring demonstrated a larger rate of either ≥ 3 mm or ≥ 5 mm displacement than the current study. This variation might occur because real-time monitoring detects transient excursion which cannot be detected with pre and post-treatment imaging. When comparing to the definitive prostate setting Cuccia et al. found that the intra-fraction median translational displacement was 0.11 mm in SI, − 0.24 mm in LR, and − 0.22 mm in AP which is smaller than what was detected in our prostate bed cohort [13].

Real-time monitoring has the advantage of being able to monitor intra-fraction motion of the prostate bed throughout the entire treatment which allows for more accurate evaluation and intervention to correct for the motion which was not possible using the pre and post-treatment CBCT method. However, one of the advantages of the pre and post-treatment CBCT method used in our study was that the soft tissue could be visualised and differences readily quantified. Prostate bed IFD is difficult to assess because the surgical bed both deforms and moves in a translational manner. Previous research has shown that the prostate bed deforms with larger motion detected in the upper portion of the prostate bed compared to the lower [3]. The accuracy of real-time monitoring depends on the placement of surrogates into the areas of the prostate bed and their ability to predict the way it moves and deforms.

Theoretically, marginal miss would most commonly occur if there was an IFD ≥  ± 5 mm due to the PTV margins used; however only 5.4% of the all fractions demonstrated a 3D displacement in this range and only 29% of the images that detected a marginal miss had an 3D displacement IFD ≥  ± 5 mm. Marginal miss may occur with even very small amounts of IFD due to deformation of the prostate bed, with the upper portion more susceptible to inter-fraction motion than the lower portion [3], which can lead to the tumour bed being aligned at the edge of the PTV during the pre-treatment CBCT soft tissue match. The resulting close margin means even 2 mm of motion may cause a miss. It is difficult to translate this information directly to the PTV margins used because of the deformation that is seen each fraction. This suggests that further investigation into adequate PTV margins or a tolerance system for online matching is required for these patients which is the focus of ongoing research.

The location and size of the marginal misses of the prostate bed was also assessed. Most misses occurred in the upper central portion of the prostate bed where the CTV is located between the bladder and rectum. It is interesting to note that the marginal misses were relatively small with 77% being ≤ 3 mm in size. Larger misses of > 5 mm were only detected on 11% of the images. It is also interesting to note that all of the lymph node and combined prostate bed and lymph node marginal misses occurred in the first five fractions but the use of the first 5 fractions as a predictor for further prostate bed marginal misses failed with many of the patients recording a marginal miss in the first 5 fractions not having further miss or patients who did not record a miss in the first 5 fractions recording misses later in the treatment course. This highlights the random nature of IFD and suggests that real-time intra-fraction monitoring or daily adaptive radiotherapy may well be of benefit to these patients. Daily adaptive radiotherapy for definitive prostate patients using a magnetic resonance linear accelerator (MR-linac) has been demonstrated to be a feasible and accurate way of treating these patients when compared to IGRT treatments which means that adaptive radiotherapy might also benefit prostate bed patients. [14]

It is important to note that our study included 46 patients which, to our knowledge, is the largest patient cohort to date with the previous highest being 20 patients [8, 9]. The only other studies that used CBCT scans to quantify intra-fraction motion included 14—18 patients [6, 7]. Our study is also unique because, to our knowledge, it is the only study that has evaluated soft tissue and/or surgical clip marginal miss and its location. Some have used excursions > 3 mm or 5 mm as a surrogate but none have evaluated marginal miss using a soft tissue detection method.

Lymph nodes were treated in 25 (54.3%) of the 46 patients in this study. During review it was noted that the prostate bed can move independently to the lymph node volumes. This has been reported previously in definitive prostate radiotherapy [15]. Our matching technique priorities matching to the prostate bed and checking that the soft tissue delineated in the lymph node CTV on the planning CT scan is included in the lymph node PTV on the CBCT scan. The varied motion means it can be difficult to align both the prostate bed and lymph node and a compromised match might be required. This leads to a larger risk of IFD causing marginal miss. Careful consideration of the CTV to PTV expansion on the lymph node volumes is needed. This is especially important when gross tumour volumes (GTV) are located in the lymph nodes. A PTV margin is required around the GTV that allows accurate alignment when the primary match is made to the prostate bed. In this group of patients only 9 had a GTV nodal boost and most of these had an adequate PTV expansion around the GTV to ensure averaged matching was minimised.

**Fig. 6:**
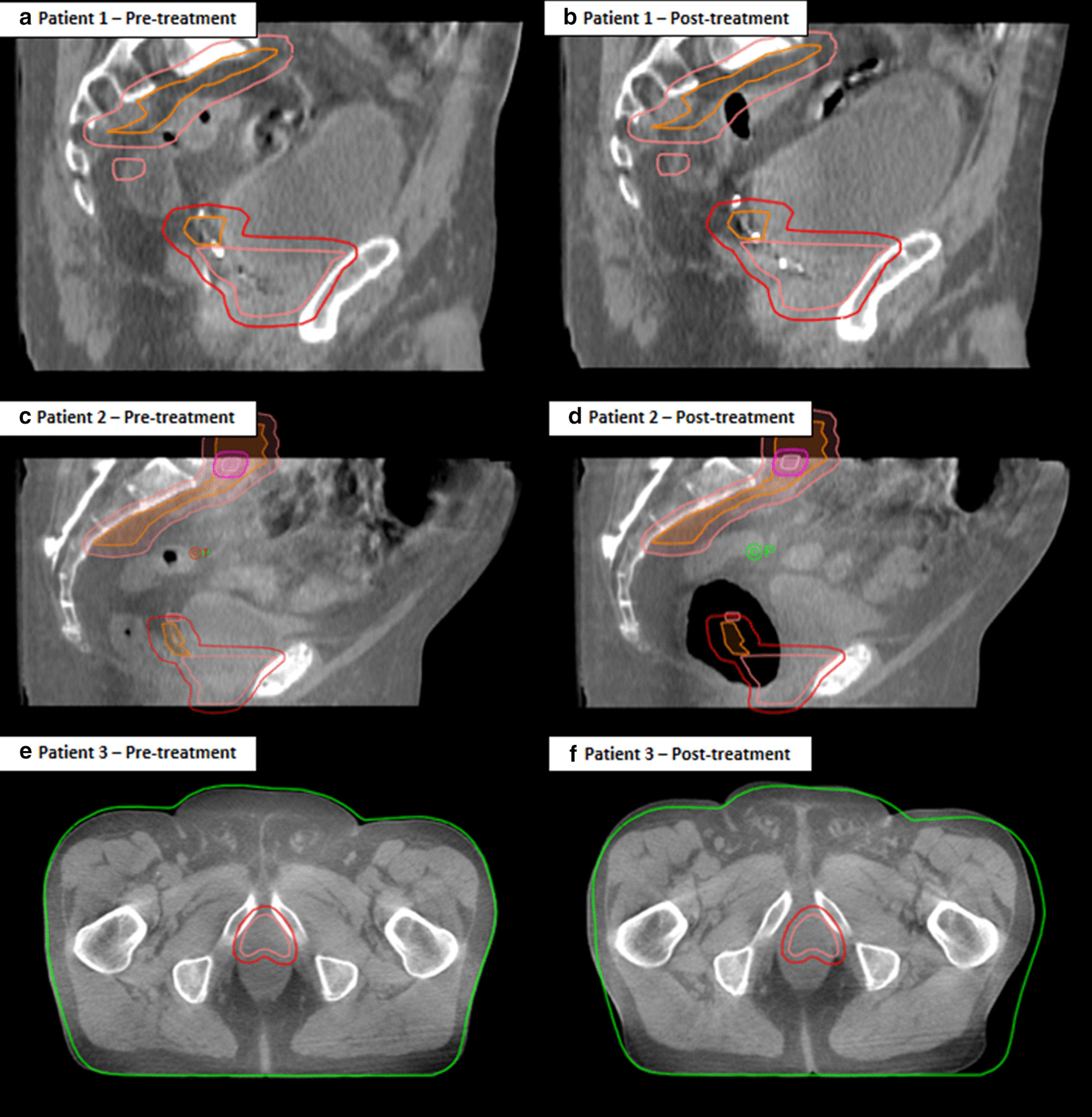
Images showing intra-fraction displacement. The pre (**a, c, e**) and post-treatment (**b, d, f**) CBCT scans for 3 patients are displayed. In patient 1′s pre-treatment **a** the superior clip in within the prostate bed PTV (red volume) but on the post-treatment **b** the bladder had continued to fill by a small amount and caused the prostate bed and superior clip to move posterior and superior causing a marginal miss. Patient 2’s pre-treatment **c** had little rectal gas present but on the post-treatment **d** a large amount of gas appeared in the prostate bed region which caused the prostate bed to move anteriorly resulting in a marginal miss. The third patient’s pre-treatment **e** showed relaxed buttock check and in the post-treatment **f** the left buttocks cheek had tensed after the bladder continued to fill and caused the prostate bed to move laterally

Some of the most common causes of IFD seen while assessing the pre and post-treatment CBCT are displayed in Fig. 6. In patient 1’s pre-treatment image (a) the superior clip and prostate bed were within the prostate bed PTV (red volume) but on the post-treatment image (b) the bladder had continued to fill, the prostate bed and superior clip moved posterior and superior leading to a marginal miss. This demonstrates that even small changes in bladder size can cause IFD which lead to a marginal miss. The second patient’s pre-treatment image (c) had minimal rectal gas, however on the post-treatment image (d) rectal gas has moved and caused the prostate bed to shift anteriorly, leading to a marginal miss. The third patient’s pre-treatment image (e) showed relaxed buttock checks, but in the post-treatment image (f) the left buttocks cheek had tensed after the bladder continued to fill moving the prostate bed laterally. In many cases the pre-treatment CBCT image gave little indication of the change in patient anatomy that occurred during treatment. Bladder filling in the time between the pre-treatment and post-treatment CBCT acquisition occurred in 60.6% of the images where a marginal miss was detected. Therefore caution needs to be taken when hydrating patients for treatment. Adequate time needs to be given between finishing drinking the water for treatment preparation and the start of treatment delivery to ensure bladder filling stabilises. An increase in the time between the pre to post-treatment CBCT was also associated with an increase in IFD, and marginal miss indicating shorter treatment times with VMAT or fast IMRT (e.g. Halcyon) should be considered for post-prostatectomy patients, particularly if margins are reduced.

There are a number of limitations with this study. The use of pre and post-treatment CBCT for IFD, while being accessible, only provides a snapshot in time of IFD. This type of motion detection is not real-time and only shows the start and end position of the target on images that are acquired before and after treatment delivery with no information about what happened between these 2 time points. Correction of IFD was also not possible using this technique. Another limitation was that post-treatment CBCT images were not taken at every treatment fraction. This means that not all IFD was measured over the entire course of treatment which could lead to an under or over estimation of motion. A marginal miss in our study was defined as any soft tissue and/or surgical clips contoured within the CTV on the planning CT scan being located outside the PTV on the CBCT. On many occasions the amount of tissue located outside the PTV was minimal suggesting little if any clinical significance. To reinforce this argument, a PSMA-PET study previously completed at our institution identified sites of failure in men with rising PSA after post-prostatectomy radiotherapy treatment. Only 3 of the 67 men with PSA failure following post-prostatectomy radiotherapy who underwent PSMA scanning demonstrated in-field failure [16]. This study has not evaluated the dosimetric effect that IFD has on surrounding critical structures such as the bladder and rectum and has focused solely on target coverage. Images which displayed a marginal miss on the pre-treatment imaging due to a setup error (n = 16) were removed from the analysis. This could result in bias in the IFD measurements but it was felt that removing these images would result in a more accurate evaluation.

The findings that small amounts of IFD can cause marginal miss, and the difficulties of calculating PTV margins that account for daily deformation has led to our future research utilising adaptive radiotherapy to correct for prostate bed deformation daily. Being able to start treatment with GTV and CTV contours tailored to the patient at the time of treatment could decrease the effect of IFD on marginal miss and allow for reduced PTV expansions and possibly even hypofractionated courses of post-prostatectomy radiotherapy.

## Conclusions

IFD during prostate bed irradiation was generally small but was a major contributor to an 8.4% marginal miss rate when using daily soft tissue match and tight anisotropic margins especially when treatment time was prolonged. Allowing 2–3 mm for IFD needs to be incorporated for PTV expansions and correction strategies.

## Supplementary Information

**Additional file 1**. Prostate bed intra-fraction motion comparison.

## Data Availability

The datasets used and/or analysed during the current study are available from the corresponding author on reasonable request.

## Abbreviations

IFD: Intra-fraction displacement
CBCT: Cone beam computed tomography
PB: Prostate bed
MM: Marginal miss
IGRT: Image guided radiotherapy
PTV: Planning target volume
VMAT: Volumetric modulated arc therapy
IMRT: Intensity modulated radiotherapy
AP: Anterior–posterior
SI: Superior–inferior
LR: Left–right
CTV: Clinical target volumes
