# Supplementary material for: Intra-fraction displacement of the prostate bed during post-prostatectomy radiotherapy
Source: Radiat Oncol. 2021 Jan 22;16:20. doi: 10.1186/s13014-020-01743-9 (PMC7821719; doi:10.1186/s13014-020-01743-9)
Supplement: Supplementary file 1 — Additional file 1. Prostate bed intra-fraction motion comparison. [file 13014_2020_1743_MOESM1_ESM.pdf]

Additional file 1 – Prostate bed intra-fraction motion comparison.

Comparison of prostate bed motion in this study compared with previously published data.

Author Name	Imaging Type	Number of patients (number images)	Measurement of intra-fraction motion
Current Study	CBCT<br>(pre and post-treatment)	46 (412 post-treatment images)	***Median (Range)***<br>Anterior 1.0 mm (0.1 mm to 19.1 mm), posterior 1.2 mm (0.1 mm to 8.4 mm), superior 1.3 mm (0.1 mm to 5.6 mm), inferior 1.0 mm (0.1 mm to 3.6 mm), left 0.7 mm (0.1 mm to 3.7 mm), right 0.9 mm (0.1 mm to 6.5 mm), and 3D displacement 2.1 mm (0 mm to 19.3 mm).
			Absolute Mean (95% CI)<br>AP 1.5 mm (1.3 mm to 1.6 mm), SI 1.0 mm (0.9 mm to 1.2 mm), LR 0.8 mm (0.7 mm to 0.9 mm), and 3D displacement 2.4 mm (2.2 mm to 2.5 mm)
			Displacement<br>• Motion ≥ 3 mm<br>Any motion (AP, SI, LR) 20.9% of fractions, AP motion 9.9% of fractions, SI motion 6.4% of fractions, LR motion 4.6% of fractions, 3D displacement 24.7% of fractions<br>• Motion ≥ 5 mm<br>Any motion (AP, SI, LR) 3.6% of fractions, AP motion 2.0% of fractions, SI motion 1.0% of fractions, LR motion 0.5% of fractions, 3D displacement 5.4% of fractions
Klayton et al [8]	Calypso	20 (638)	***Displacement***<br>• 15% of all treatments were interrupted for repositioning<br>• 70% of patients repositioned at least once during their treatment course<br>• 90% of patients exceeded the 5 mm threshold at least once during treatment course
			Time<br>• 5 mm tracking limited exceeded ≥ 1 sec (occurred in 32% of 638 fractions)<br>AP 159 fractions (25%), SI 176 fractions (28%), LR 10 fraction (1.6%)<br>• 5 mm tracking limited exceeded ≥ 30 secs (of 638 fractions)<br>AP 31 fractions (5%), SI 44 fractions (7%), LR 1 fraction (<0.5%)<br>• Target excursion ≥ 10 mm (of 638 fractions)

			AP 13 fractions (2%), SI 20 fractions (3%), LR 0 fractions (0%)<br>• Area under curve ≥1 (of 487 fractions)<br>AP 30 fractions (6%), SI 39 fractions (8%), LR 1 fraction (<0.5)
Huang et al [6]	CBCT<br>(after setup, after shift and after treatment)	14 (140 post-treatment images)	***Prostate Bed Motion Overall Mean***<br>AP 0.2 mm, SI -0.4 mm, LR 0.1 mm
			Prostate Bed Motion Standard Deviation of random error<br>AP 3.1 mm, SI 2.8 mm, LR 2.0 mm
			Prostate Bed Motion Standard Deviation of systematic error<br>AP 1.1 mm, SI 1.0 mm, LR 0.7 mm
			Magnitude of mean overall motion Prostate Bed Motion<br>0.4 mm
Fargier-Voiron M et al [11]	Ultrasound	7 (124)	***Displacement***<br>• 35% of fractions have shifts above 3 mm in AP direction (this result combines both definitive prostate and post-prostatectomy patients)
Fargier-Voiron M et al [12]	Ultrasound	14 (438)	***Displacement***<br>• During 1st min, displacement > 3 mm = 1.9% of the time<br>• After 7 mins of treatment, displacement 3 mm = 10.8%<br>• Max 3D displacements > 5 mm after 7 mins = 1.6% of the time
			Time<br>• 3 mm tracking limit exceeded = 15 secs<br>AP 11.4% of sessions, SI 0.7% of sessions, LR 4.1% of sessions<br>• 3 mm tracking limit exceeded = 30 secs<br>AP 9.4% of sessions, SI 0.7% of sessions, LR 3.4% of sessions<br>• 5 mm tracking limit exceeded = 15 secs<br>AP 1.8% of sessions, SI 0% of sessions, LR 0.7% of sessions<br>• 5 mm tracking limit exceeded = 30 secs<br>AP 1.6% of sessions, SI 0% of sessions, LR 0.7% of sessions
			Mean Displacement

			AP 0.9 ± 0.5 mm, SI 0.2 ± 0.4 mm, LR 0.1 ± 0.4 mm
King et al [9]	Calypso	20 (695)	***Displacement***<br>• Maximum motion<br>Left = 5.6 mm, Right = 10.2 mm, Superior = 16.4 mm, Inferior = 9.2 mm, Anterior = 17.9 mm, Posterior = 9.6 mm<br>• Displacement >3 mm occurred in 57% of fractions<br>• Displacement >5 mm occurred in 19% of fractions<br>***Time***<br>• Mean total treatment cumulative time outside 3 mm = 606 secs (range 8 secs-2401 secs) during tracking<br>• Which was reduced to 40.5 secs (1secs-134 secs) during beam-on time
Foster et al [10]	Calypso	9 (320)	***Displacement***<br>• Displacement >3 mm occurred in 8.8% (range 4.3% to 18.8%)of fraction<br>• Displacement >5 mm occurred in 0.86% (range 0.25 to 1.76%)of fraction<br>***Time***<br>• SI direction only<br>>3 mm = 2.30% of tracking time, >5 mm = 0.42% of tracking time, >10 mm = 0.04% of tracking time
Yoon et al [7]	CBCT<br>(pre and mid-treatment)	18 (175)	***Mean Displacement***<br>AP 1.1 mm, SI 2.3 mm, LR 2.0 mm, Rotation 0.6

Abbreviations: 3D = 3 dimensional, mm = millimetre, CBCT = cone beam computed tomography, mins = minutes, secs = seconds, AP = anterior – posterior, SI = superior – inferior, LR = left – right,
